# Advancing nursing practice with artificial intelligence: Enhancing preparedness for the future

**DOI:** 10.1002/nop2.2070

**Published:** 2023-12-20

**Authors:** Moustaq Karim Khan Rony, Mst. Rina Parvin, Silvia Ferdousi

**Affiliations:** ^1^ Master of Public Health Bangladesh Open University Gazipur Bangladesh; ^2^ Major of Bangladesh Army Combined Military Hospital Dhaka Bangladesh; ^3^ International University of Business Agriculture and Technology Dhaka Bangladesh

**Keywords:** artificial intelligence, future, healthcare, nursing practice, patient outcomes, preparedness

## Abstract

**Aim:**

This article aimed to explore the role of AI in advancing nursing practice, focusing on its impact on readiness for the future.

**Design and Methods:**

A position paper, the methodology comprises three key steps. First, a comprehensive literature search using specific keywords in reputable databases was conducted to gather current information on AI in nursing. Second, data extraction and synthesis from selected articles were performed. Finally, a thematic analysis identifies recurring themes to provide insights into AI's impact on future nursing practice.

**Results:**

The findings highlight the transformative role of AI in advancing nursing practice and preparing nurses for the future, including enhancing nursing practice with AI, preparing nurses for the future (AI education and training) and associated, ethical considerations and challenges. AI‐enabled robotics and telehealth solutions expand the reach of nursing care, improving accessibility of healthcare services and remote monitoring capabilities of patients' health conditions.

## 1 INTRODUCTION

With the advent of artificial intelligence (AI), nursing practice will advance in the modern healthcare landscape (Ronquillo et al., 2021). AI has the potential to revolutionize healthcare delivery, improve patient outcomes, and transform the role of nurses (Clancy, 2020). Embracing AI can greatly enhance nursing practice in several key areas. First, AI‐powered clinical decision support systems can provide nurses with valuable insights and evidence‐based recommendations, helping them make informed decisions about delivering patient care (Van Bulck et al., 2023). AI can help nurses diagnose and treat patients more effectively with higher precision by analysing extensive amount of evidence and seeing similarities (Buchanan et al., 2020a, 2020b). Second, AI can enhance patient monitoring and support predictive analytics. Moreover, through AI‐enabled technologies, nurses have the capability to continuously monitor patients' vital signs, detect early warning signs of deterioration and receive real‐time alerts (Alazzam et al., 2022). This allows for timely interventions, reduce the risk of adverse events and improve patient outcomes (McGrow, 2019). Third, AI has the potential to streamline care coordination and reduce the nursing workload. AI algorithms can automate administrative tasks, prioritize patient needs and facilitate seamless communication in a healthcare team (Stokes & Palmer, 2020). This enables nurses to focus more on direct patient care and ensures the efficient and coordinated delivery of healthcare services (Barrera et al., 2020). Furthermore, advancing nursing practice in the context of AI requires nurses to continuously acquire new knowledge and skills (Fritz & Dermody, 2019). A nurse's ability to use AI will be greatly enhanced if AI learning and instruction are incorporated into the nursing curriculum (Dermody & Fritz, 2019). This will empower them to adapt to technological advancements, embrace innovation and provide high‐quality care in the digital era (Hegde et al., 2022).

The integration of AI into the nursing profession is ushering in new opportunities and advancements in patient care (Pepito & Locsin, 2019). Nurses stand to benefit significantly from the adoption of AI technologies in various healthcare settings. AI plays a pivotal role in aiding nurses in making medical judgements. It accomplishes this by analysing extensive patient data, research findings, and medical literature through natural language processing and machine learning, thereby facilitating evidence‐based decision‐making (Rubeis, 2020). The utilization of AI promises to enhance the quality and accuracy of nursing diagnoses and intervention plans by providing nurses with real‐time information and evidence‐based recommendations (Randhawa & Jackson, 2020). Furthermore, AI‐driven virtual assistants can assist in patient education by delivering information on medications and self‐care practices, as well as addressing commonly asked questions (Bays et al., 2023). Additionally, nurses can take proactive measures with the support of AI‐based anticipatory analytics, which helps in identifying potential patient deterioration or readmission risks (Booth et al., 2021). This article aimed to explore how artificial intelligence (AI) can bolster nurses' preparedness for the future, equipping them with the skills and tools necessary to navigate the evolving healthcare landscape while empowering them to provide enhanced patient care.

### 1.1 Overview of artificial intelligence in nursing

AI, or artificial intelligence, is the field of computer science that focuses on developing intelligent machines capable of performing tasks that typically require human intelligence (Pepito et al., 2020). In healthcare, algorithms and data analysis techniques used by AI help analyse medical data, aid in therapeutic decision‐making, automate management procedures, increase patient tracking and better coordinate treatment process and steps (Jacques et al., 2021). AI applications such as machine learning, natural language processing (NLP), computer vision (CV) and statistical prediction are included to improve healthcare outcomes and remodel healthcare delivery (Lee & Yoon, 2021).

AI applications in nursing practice span a broad spectrum of areas. For instance, AI‐driven clinical decision support systems stand out as an example. These systems analyse patient data, offer evidence‐based recommendations and assist nurses in achieving precise diagnoses and treatment plans (Jain et al., 2021; Lynn, 2019). Moreover, AI finds its place in patient monitoring as well, with algorithms continuously scrutinizing vital signs, identifying patterns, and notifying nurses of potential changes or deteriorations (Ng et al., 2022). Additionally, AI holds promise in reducing the burden of paperwork, enhancing care delivery and fostering collaboration among diverse healthcare stakeholders (Jeong, 2020). These instances vividly illustrate how AI can elevate nursing practice and enhance patient outcomes.

The utilization of AI in clinical nursing brings forth several favourable outcomes. First, AI equips nurses with statistical information and evidence‐based suggestions, thus refining their decision‐making processes and contributing to more precise assessments and therapeutic strategies (Yang et al., 2022). Second, AI's role in patient surveillance facilitates rapid interventions, resulting in improved patient outcomes (Frith, 2019). Furthermore, automation through AI streamlines administrative tasks, affording nurses more time to focus on patient care (Clipper et al., 2018). AI technologies also facilitate care coordination, bolstering communication and teamwork within the medical sector (Chen et al., 2022).

## 2 METHODOLOGY

### 2.1 Literature search and selection

The initial phase of this paper involved a comprehensive and meticulously structured literature search. A range of reputable databases, including but not limited to PubMed, CINAHL, and Google Scholar, was utilized to retrieve published literature. To ensure inclusivity, specific keywords and phrases directly pertinent to the intersection of nursing practice and artificial intelligence (AI) were employed. We used Boolean operators (AND, OR, NOT) to refine our search, such as (“nursing practice” OR “clinical practice”) AND (“artificial intelligence” OR “AI” OR “future preparedness”). These keywords encompassed various facets of AI integration in healthcare, from its applications in diagnostics to its role in improving patient care. The search encompassed a diverse array of academic sources, comprising peer‐reviewed articles, conference papers, research reports and scholarly books. The search was restricted to include publications up to the present date within the last 6 years, ensuring that the review captured the latest developments and insights in this dynamic field.

To uphold the highest standards of quality and relevance, we implemented a rigorous screening process, including initial abstract reviews, comprehensive full‐text evaluations, and quality assessments. Seeking consensus among researchers ensured a thorough and meticulous selection of relevant studies. Each identified article underwent careful evaluation for its direct relevance to the central theme of the review. This rigorous selection process ensured that only articles that substantially contributed to the understanding of AI's impact on nursing practice and strategies for nurses' preparedness were retained for further analysis.

### 2.2 Data extraction and synthesis

The subsequent phase of our research involved the critical process of data extraction, focusing on studies that directly explored the integration of artificial intelligence (AI) into nursing practice and its implications for future preparedness. A meticulous approach guided the inclusion of studies aligning with the objectives of this position paper. From each selected article, we carefully extracted information crucial to our goals. This encompassed a wide spectrum, from delineating the multifaceted roles of AI in nursing practice to examining its measurable impact on patient care outcomes. Additionally, we delved into the identification of obstacles and challenges encountered in the integration of AI within the nursing domain. Furthermore, the exploration extended to encompass the diverse strategies proposed for enhancing nurses' preparedness in embracing AI technologies.

The data extracted form a comprehensive and diverse dataset, capturing the nuanced dimensions of AI's involvement in nursing. These individual data points, meticulously collected, were then skillfully aggregated and synthesized. Through this process, we transformed disparate pieces of information into coherent and structured themes, providing a unified understanding of the intricate relationship between AI and nursing practice, paving the way for future readiness.

### 2.3 Thematic analysis

The synthesis of data was followed by a rigorous thematic analysis, a critical component of this review. The goal was to identify recurring themes, patterns and significant trends that emerged across the reviewed literature. By discerning these common threads, a structured overview of AI adoption within nursing practice was constructed. The thematic analysis allowed for the distillation of the essence of the reviewed literature, presenting readers with a clear and nuanced understanding of how AI is impacting the field of nursing and healthcare at large. This comprehensive approach to data analysis not only enabled the clarification of the many‐sided nature of AI's role in nursing but also provides a vigorous foundation for further discussions and insights. Through this rigorous methodology, valuable contributions to the ongoing discourse on AI integration in nursing practice and the strategies required to enhance nurses' preparedness for an AI‐driven future in healthcare were aimed to be offered.

## 3 ENHANCING NURSING PRACTICE WITH AI

### 3.1 AI‐powered clinical decision support systems

Clinical decision‐making stands to profit significantly from the application of AI in healthcare. Medical records, laboratory results and imaging tests are just some types of patient data that AI‐powered systems may analyse to deliver insights and recommendations based on science to healthcare professionals (Buchanan et al., 2020a, 2020b). This allows for more precise diagnoses, tailored treatment strategies and enhanced individual health outcomes. Clinical planning can be aided by AI algorithms' ability to recognize patterns, flag outliers and anticipate hazards (Maddox et al., 2019). Furthermore, artificial intelligence's ability to rapidly and effectively process vast amounts of information can lead to enhanced precision, performance and responsiveness to the unique needs of individual patients in the clinical setting (Watson et al., 2020).

Artificial intelligence (AI) has the potential to enhance nurses' knowledge and lead to better health outcomes for their patients. By sifting through mountains of information, AI can help nurses arrive at more precise diagnoses and care plans (McGreevey et al., 2020). Nurses' clinical judgement can be improved with the help of AI‐powered systems that provide evidence‐based suggestions, recognize trends in patient data, and provide insights (Noorbakhsh‐Sabet et al., 2019). This aids in the determination of risks, the forecasting of outcomes, and the creation of individualized treatment strategies. AI can also enhance patient monitoring by enabling the discovery of subtle changes or deterioration at an earlier stage (Matheny et al., 2020). Over time, AI has the potential to assist nurses in enhancing patient outcomes by empowering them to deliver treatment that is more accurate, efficient, and patient‐centered (McCall, 2020). AI algorithms' success is highly dependent on the quality and representativeness of the data used for training, making it challenging to guarantee their accuracy and reliability (Parikh et al., 2019). In addition, concerns about data privacy, security and patient consent must be resolved to safeguard private medical data. Transparency in AI decision‐making continued human monitoring, and the promotion of equitable access to AI technologies are all moral issues (Emanuel & Wachter, 2019).

### 3.2 AI‐enabled patient monitoring and predictive analytics

Many healthcare benefits can be directly attributed to AI‐driven patient monitoring systems. Using AI algorithms, these systems constantly analyse patient data, such as health indicators, laboratory results, and data from wearable devices (Khan & Alotaibi, 2020). AI can monitor a patient's status and spot patterns, abnormalities, and changes with the help of advanced analytics and machine learning (Mehta et al., 2019). This allows for early detection of any deterioration or consequences and fast treatment. Patient safety is increased, proactive and individualized care is enabled, and the likelihood of adverse occurrences is decreased using AI‐driven monitoring systems (Abidi & Abidi, 2019). They equip medical professionals with helpful information that ultimately benefits patient outcomes and treatment quality.

AI‐powered predictive analytics prove valuable for the early recognition of signs indicating patient decline. These algorithms can identify subtle changes or emerging trends that may hint at a patient's deteriorating condition by scrutinizing historical patient data alongside real‐time monitoring information (Van Calster et al., 2019). Swift detection empowers medical professionals to respond promptly, mitigating adverse outcomes. The use of predictive analytics also allows nurses to optimize care prioritization and resource allocation, ultimately leading to enhanced patient welfare. Proactive care guided by predictive analytics contributes to reduced hospital readmissions, improved patient flow and heightened safety levels (Rosenfeld et al., 2021).

When it comes to using AI for preventative care, nurses are crucial players. They play a pivotal role in patient interactions and bring invaluable clinical knowledge. Analysing massive amounts of patient data, like clinical records and continuous surveillance data, can help nurses detect patterns, anticipate risks and take precautionary steps (Secinaro et al., 2021). AI driven decision support systems have the capacity to assist nurses in enhancing patient outcomes by improving diagnosis, treatment, care planning and optimizing resource utilization (Davenport & Kalakota, 2019). Together, nurses and AI algorithms can track patients' vitals, identify potential danger indicators and provide prompt care. Patient‐centred treatment, better outcomes, and increased patient safety can all be achieved when healthcare providers actively employ AI tools (Richardson et al., 2021).

### 3.3 AI‐assisted care coordination and workload management

AI algorithms offer the potential to streamline care coordination processes in healthcare. Automatic appointment scheduling and resource allocation are only two examples of managerial tasks that can benefit from AI's computerization (Banerjee et al., 2020). AI‐powered care coordination platforms enable seamless communication and collaboration among healthcare teams, improving information sharing and reducing delays (Hassan et al., 2021). These algorithms can analyse patient data, identify care gaps and provide recommendations for appropriate interventions, facilitating coordinated and personalized care delivery (Chen & Decary, 2020). Streamlining care coordination processes with AI algorithms enhances communication, minimizes errors and improves patient outcomes (Montella et al., 2022).

Tools powered by AI can streamline the distribution of nurse workloads, guaranteeing resource allocation that is both efficient and equitable. Through AI algorithms, factors like patient acuity, workload and staffing levels are analysed to determine the most suitable task assignments (Bohr & Memarzadeh, 2020). AI tools can balance workloads across the nursing team by considering factors like patient complexity, nurse expertise and time‐sensitive needs (Burton et al., 2019). This helps prevent burnout, reduces the risk of errors and improves the overall quality of care. Optimizing nurse workload distribution using AI‐driven tools enhances job satisfaction, allows nurses to focus on direct patient care and ultimately improves patient outcomes (Kwee & Kwee, 2021).

Integrating AI in healthcare has significant implications for improved patient safety and nursing efficiency. AI‐powered systems can analyse vast amounts of patient data, identify patterns, and detect anomalies, enabling early identification of potential risks or adverse events (Choudhury & Asan, 2023). By providing real‐time alerts and predictive analytics, AI enhances patient safety by facilitating timely interventions and preventive measures. Additionally, with the help of AI technology, nurses may spend less time on documentation and more time providing hands‐on care to patients (Dembrower et al., 2020). This improves nursing efficiency, enhances workflow, and promotes a safer and more effective healthcare environment.

## 4 PREPARING NURSES FOR THE FUTURE: AI EDUCATION AND TRAINING

### 4.1 Integrating AI education into the nursing curriculum

Nursing professionals can derive significant advantages from gaining a solid grasp of fundamental AI concepts and cultivating pertinent skills. These encompass an understanding of machine learning, natural language processing, data analysis and AI algorithms (Chang et al., 2022). Equally vital is familiarity with AI ethics, privacy and security considerations. Furthermore, nurses should possess strong critical thinking skills, enabling them to interpret AI‐generated insights and effectively apply them to enhance patient care (Sitterding et al., 2019). Understanding AI's limitations, potential biases and the importance of human oversight is essential. By acquiring these core AI concepts and skills, nursing professionals can effectively utilize AI tools, make informed decisions and contribute to improved patient outcomes (Ahmad & Jenkins, 2022). Incorporating AI education into existing nursing programs requires careful planning and implementation. Strategies include designing dedicated AI courses or modules that cover core AI concepts, applications in healthcare and ethical considerations (Von Gerich et al., 2022). Integrating practical experiences, such as hands‐on AI tool usage or case studies, can enhance understanding. Collaboration with AI experts and industry partners can provide valuable insights and resources (Harmon et al., 2021). Promoting continuous learning through the provision of ongoing education opportunities and motivating engagement in AI‐related conferences or workshops is essential. It is crucial to maintain flexibility in adapting the curriculum to accommodate the ever‐evolving AI technologies, ensuring that nursing professionals remain well‐prepared to leverage the advantages of AI in their practice (Liaw et al., 2023).

Collaborative efforts between academia and healthcare institutions are crucial for effectively incorporating AI education into nursing programs. Partnerships can facilitate sharing of expertise, resources, and best practices. Joint initiatives can include developing AI curriculum guidelines, establishing simulation labs, or creating clinical placements in AI‐focused settings (Hwang et al., 2022). Collaborative research projects can explore AI's impact on nursing practice and patient outcomes. Continuous communication and feedback between academia and healthcare institutions ensure educational programs align with industry needs and emerging AI advancements (Liao et al., 2015). In addition, nursing personnel can help improve patient care by incorporating AI if academic and medical institutions collaborate to train them for the new healthcare scenario.

### 4.2 Continuous professional development on AI and emerging technologies

**FIGURE 1:**
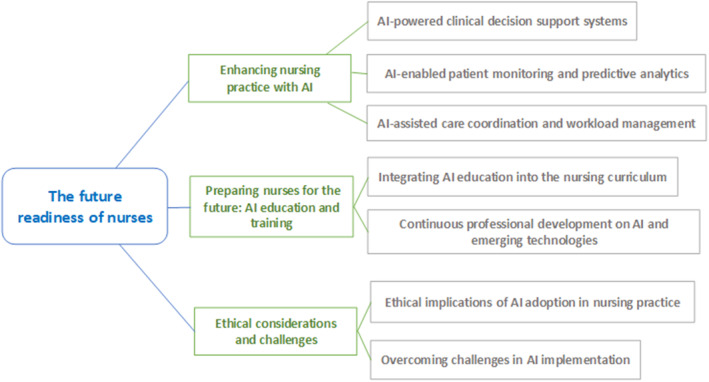
Nurses preparedness for the future.

Ensuring nurses remain well‐informed about the swift advancements in AI (Figure 1) is vital for lifelong learning. Nurses can actively participate in ongoing professional development programs and partake in workshops, webinars, or conferences focused on AI in healthcare (Randhawa & Jackson, 2020). The flexibility of self‐paced learning is readily accessible through online platforms and resources, including AI‐oriented courses and educational websites. Collaborating with AI experts and interdisciplinary teams can provide invaluable insights (Abuzaid et al., 2022). Facilitating knowledge sharing and keeping abreast of developments via professional networks and journals is key to ensuring that nurses are continuously informed and equipped to leverage the latest AI innovations in their practice. To equip nurses with AI skills, dedicated training programs and resources are essential (O'Connor et al., 2022). These can encompass structured courses or workshops covering AI fundamentals, applications in nursing practice, and hands‐on training with AI tools. Online modules, e‐learning platforms or mobile apps offer accessible, self‐directed learning opportunities (Ronquillo et al., 2021). Collaborative efforts with universities, industry partners and professional associations can facilitate the creation of comprehensive training programs (Ahuja et al., 2023). Furthermore, mentorship programs and shadowing experiences with AI experts can enhance practical knowledge and skill development for nurses looking to integrate AI into their practice (He et al., 2019).

Creating a culture of technological proficiency in nursing practice involves fostering an environment that embraces and encourages the use of technology, including AI. This can be achieved through leadership support, promoting technological literacy and providing resources for training and upskilling. Nursing graduates who have been taught to use technological tools effectively are more likely to be successful in their careers (Harmon et al., 2021). Encouraging nurses to participate in technology‐related committees or quality improvement projects fosters innovation (Tang et al., 2021). Collaborative efforts between nurses, IT professionals and administrators promote knowledge exchange and interdisciplinary collaboration, resulting in a culture of technical proficiency that embraces AI and other advancements in healthcare technology.

## 5 ETHICAL CONSIDERATIONS AND CHALLENGES

### 5.1 Ethical implications of AI adoption in nursing practice

Protecting patients' identities and medical records should be the top priority for AI‐powered healthcare systems. Encryption, access controls, and secure data storage are essential to safeguard patient information (Pruinelli & Michalowski, 2021). Compliance with data protection regulations, such as health insurance portability and accountability act (HIPAA), is crucial. Transparent policies and informed consent processes should be in place to ensure patients know how their data are used (Tabudlo et al., 2022). Regular audits and risk assessments can identify vulnerabilities and enhance security protocols. To build trust with patients and ensure the responsible use of AI, healthcare providers should prioritize the protection of patients' personal information and health records (Aung et al., 2021).

Transparency and accountability are vital when implementing AI algorithms and decision‐making in healthcare. Understanding how AI algorithms reach their conclusions and make decisions is crucial. Transparency can be achieved by providing explanations and justifications for AI‐generated insights (Keskinbora, 2019). Organizations should ensure that AI algorithms are continuously monitored and validated for accuracy and fairness. Ethical guidelines and industry standards should be followed to ensure the responsible use of AI (Wani et al., 2022). Transparent and accountable AI algorithms foster trust among healthcare professionals and patients, promoting the acceptance and adoption of AI‐driven technologies.

AI applications must be carefully designed and implemented to address biases and potential disparities. Biases can be unintentionally introduced into AI algorithms if the training data is not diverse or representative (Saw & Ng, 2022). Organizations must ensure that the data used for training is inclusive and avoids reinforcing existing biases. Regular audits and ongoing monitoring are necessary to identify and mitigate any biases or disparities in AI applications. Collaborating with diverse teams during AI development can bring different perspectives and help identify potential biases (Surovková et al., 2023). Healthcare organizations, through proactive measures to tackle biases and disparities, can foster fair and impartial AI applications that enhance healthcare outcomes for everyone.

### 5.2 Overcoming challenges in AI implementation

The reluctance of healthcare professionals to embrace AI can be attributed to several factors, including apprehensions about potential job displacement, limited familiarity with AI technologies and concerns regarding their reliability and accuracy. Effectively addressing these concerns necessitates the implementation of educational initiatives, training programs and transparent communication that highlights both the benefits and limitations of AI (Liyanage et al., 2019). Furthermore, involving nurses in the decision‐making process, providing robust support during the transition and presenting compelling examples of successful AI utilization can play a pivotal role in alleviating resistance and nurturing a culture of acceptance and collaboration (Li et al., 2022).

Technical barriers and interoperability issues can impede the implementation of AI in healthcare. Challenges may arise due to incompatible systems, lack of standardized data formats, or limited interoperability between healthcare IT systems (Saheb et al., 2021). Overcoming these barriers requires establishing interoperability standards, promoting data exchange protocols and investing in robust infrastructure. Collaboration between technology vendors, healthcare organizations, and regulatory bodies is crucial to address technical challenges and ensure seamless integration of AI tools into existing healthcare systems (Linnen et al., 2019).

Successful implementation and acceptance of AI tools in healthcare require careful planning and implementation strategies. These may include conducting pilot projects, engaging early adopters, and gathering feedback to fine‐tune AI algorithms and workflows. Collaboration between IT departments, clinicians and other stakeholders is essential to ensure alignment with user needs and organizational goals (Thurzo et al., 2023). Offering comprehensive training programs, providing ongoing support and showcasing the benefits of AI in improving patient outcomes can foster acceptance among healthcare professionals (Malycha et al., 2022). Continuous evaluation, data‐driven quality improvement and a culture of innovation promote successful implementation and long‐term acceptance of AI tools in healthcare.

## 6 CONCLUSION

This perspective article explored the importance of advancing nursing practice in the context of artificial intelligence (AI). We discussed the definition and scope of AI in healthcare, highlighting its potential impact on nursing practice. Examples of AI applications in nursing practice were provided, emphasizing the benefits of AI adoption, such as augmenting nurses' expertise. We also addressed potential challenges and ethical considerations in implementing AI in clinical decision support. Moreover, AI has the potential to revolutionize nursing practice and enhance patient care. With AI‐driven tools, nurses can access real‐time insights, make more accurate diagnoses, and provide personalized care plans. Artificial intelligence (AI) can aid in the timely identification of patients' declining conditions, promote the distribution of burdens, and accelerate the processes involved in care coordination. Nurses that use artificial intelligence see gains in clinical expertise, the well‐being of patients, and employee efficiency. The integration of AI in nursing practice can transform healthcare delivery and improve patient outcomes. Furthermore, nurses and healthcare institutions must embrace AI and ensure future preparedness. Nurses should engage in lifelong learning to stay updated with AI advancements, acquire core AI concepts and skills, and actively seek training programs and resources for upskilling. Healthcare facilities must prioritize patient privacy and data security in AI‐driven systems and include AI education in existing nursing student programs. Collaborative efforts between academia and healthcare institutions are essential for effective AI implementation. By embracing AI, nurses, and healthcare institutions can harness its potential to enhance nursing practice, improve patient care, and shape the future of healthcare.

## AUTHOR CONTRIBUTIONS

All authors met the contribution criteria for authorship. All authors approved the final version of the article to be published.

## FUNDING INFORMATION

There was no external fund taken for this current research.

## CONFLICT OF INTEREST STATEMENT

The authors have no competing interest at all.

## ETHICS STATEMENT

None.

## Data Availability

Data sharing not applicable to this article as no datasets were generated or analysed during the current study.
